# The complete mitochondrial genome of *Amphiesma optatum*

**DOI:** 10.1080/23802359.2020.1715873

**Published:** 2020-01-24

**Authors:** Hao Zong, Ziyong Lei, Tao Hu, Dang Feng, Jialing Li, Rong Du, Changkun Fu

**Affiliations:** College of Life Sciences, Sichuan Normal University, Chengdu, China

**Keywords:** Mitochondrial genome, *Amphiesma optatum*, phylogenetic tree, Colubridae

## Abstract

In this study, we obtained and described the complete mitochondrial genome sequence of *Amphiesma optatum*. The total length is 17,259 base pairs. Similar to most Colubridae mitochondrial genomes, there are 37 genes including 13 protein-coding genes (PCGs), 22 transfer RNA genes (tRNA), and 2 ribosomal RNA genes (rRNA). In addition, it contains two control regions (D-loop) rich in A–T base. The total base composition of mitochondrial DNA is 34.3% for A, 26.5% for C, 12.8% for G, and 26.4% for T, and the percentage of GC content is 39.3%. These data further reveal the phylogenetic relationship between *Amphiesma optatum* and other species in the Colubridae family.

*Amphiesma optatum* belongs to Colubridae family (Hu and Zhao 1966), and lives in the mountainous area with an altitude of 420–1000 m (Zhao 2003). This species is distributed in Sichuan, Guizhou, Hunan, and Guangxi provinces (Guo et al. 2012). We analyzed the sequence information and structural characteristics of the complete mitochondrial genome of *Amphiesma optatum* (Mardis 2008), and combined with the existing mitochondrial genome sequence of Colubridae family in GenBank to construct a phylogenetic tree. These results revealed the phylogenetic relationship between *Amphiesma optatum* and other species in the Colubridae family.

Specimens of *Amphiesma optatum* were collected from Mount Emei (Latitude: 29°33′57.17″N, Longitude: 103°23′31.71″E, Altitude: 827m), and are currently stored in the Zoological Museum (Specimen number: EM1906002), College of Life Sciences, Sichuan Normal University, China. In this study, the complete mitochondrial genome sequence was obtained by high-throughput sequencing method with Illumina Hiseq 2500 (Tsingke, Tianjin). The complete sequence of the mitochondrial genome was submitted to GeneBank.

The complete mitochondrial genome of *Amphiesma optatum* is 17,259 bp in length (GenBank accession number:MN427890), which contains 13 protein-coding genes (ND1, ND2, ND3, ND4, ND4L, ND5, ND6, COI, COII, COIII, ATP6, ATP8 and Cytb), 22 Transfer RNA genes (tRNA), 2 ribosomal RNA genes (rRNA), and 2 A–T base-rich control regions (D-loop). The base composition is 34.3% for A, 26.5% for C, 12.8% for G, and 26.4% for T. Like other Colubridae species, ND6 and eight tRNAs are encoded by the L-strand, whereas all the other genes are encoded by the H-strand (Wolstenholme 1992). The large ribosomal RNA (lrRNA) is 1446 bp in length and small ribosomal RNA (srRNA) is 925 bp in length. The lengths of the two control regions are 1002 bp and 1142 bp, respectively.

Based on the concatenated nucleotide sequences of protein-coding genes and two rRNAs, the phylogenetic relationships of the *Amphiesma optatum* and the other 13 snakes were constructed by MEGA6.0 using maximum-likelihood (ML) method with 1000 bootstrap replications (Tamura et al. 2013). The phylogenetic tree (Figure 1) showed that the *Amphiesma optatum* was closer to *Amphiesma vibakari* (genus *Amphiesma*) in genetic relationship, which is consistent with the results of traditional morphological classification (Guo et al. 2014). That is, the *Amphiesma optatum* belongs to the genus *Amphiesma* of the family Colubridae. This study provides data for the systematic classification of Colubridae.

**Figure 1.**
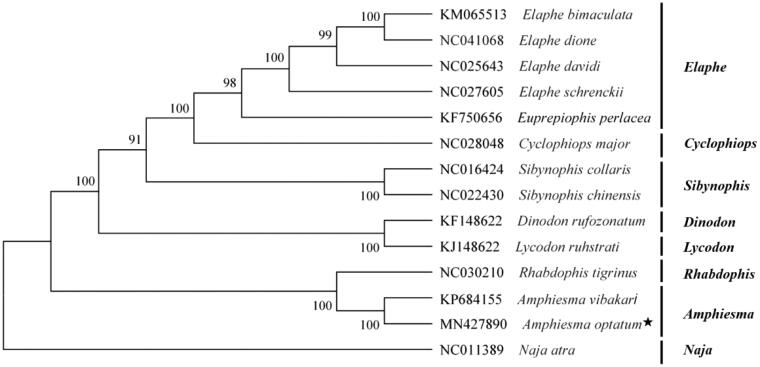
Phylogenetic tree inferred from Maximum Likelihood analysis of the nucleotide of protein-coding genes and two ribosomal RNA genes. *Naja atra* was used as outgroups. The nodal numbers indicate the bootstrap values obtained with 1000 replicates. The Genebank accession number, species name, and generic name are shown on the right side of the phylogenetic tree. The newly sequenced mitogenome is indicated by the asterisk.
